# Case of Fungous Tumor on the Back, Arising from a Scratch

**Published:** 1826-09

**Authors:** 

**Affiliations:** St. George's Hospital


### FUNGOUS TUMOR.

*Case of Fungous Tumor on the Back, arising from a Scratch.* Treated at St. George's Hospital, by Mr. Brodie.

James Bourne, ætatis fifty-four; admitted April 19th, 1826.—He has had a large cicatrix, in the situation of the present disease, between the scapulæ, for the last twenty years, originating, he says, from the severity with which he was flogged when in India. He never experienced any pain in this cicatrix, and his health was quite good, until he accidentally scratched himself with his finger-nail about four months ago; at which time the part bled, and degenerated into a sore, which continued to spread under the dressings he employed, and in a month's time the present fungus began to project, attended with severe and lancinating pain, and considerable offensive discharge. During the last three months, the tumor has increased rapidly in size; the pain and discharge have been augmented, and he has lost flesh considerably.

At the time of his admission, there was a fungus about three inches in diameter at its basis, and in some parts projecting about an inch and a half above the level of the neighbouring skin. The surface of the fungus was irregular, and divided into a multitude of small portions, giving the whole somewhat the appearance of a very large warty excrescence. The interstices of the fungus were filled with an adhesive discharge. The skin in the neighbourhood was of a dark red colour, and covered with a number of very small tubercular projections. It was thought that these might have arisen from the contact of the discharge which issued from the tumor, and therefore it was determined that the latter should be removed by operation, notwithstanding the diseased condition of the surrounding skin.

24th.—The tumor, with a small portion of the surrounding skin and a few fibres of the trapezius muscle, were removed by the knife. No considerable hemorrhage took place, and the surface was dressed with dry lint.

The wound healed rapidly, and the patient was discharged cured on June 7th; at which time the skin had regained its natural hue, and the tubercular prominences had nearly disappeared.*

* A wax cast was made of the parts described in this case by M. Delestre: it is *at present* deposited in the Museum of the College of Surgeons.

